# Supplementary material for: Novel Driver Strength Index highlights important cancer genes in TCGA PanCanAtlas patients
Source: PeerJ. 2022 Aug 11;10:e13860. doi: 10.7717/peerj.13860 (PMC9375969; doi:10.7717/peerj.13860)
Supplement: Supplemental Information 2 [file peerj-10-13860-s002.zip › COHORTS/genes plots/detailed/2021_8_16_14_9_distribution_events_detailed_males_23.pdf]

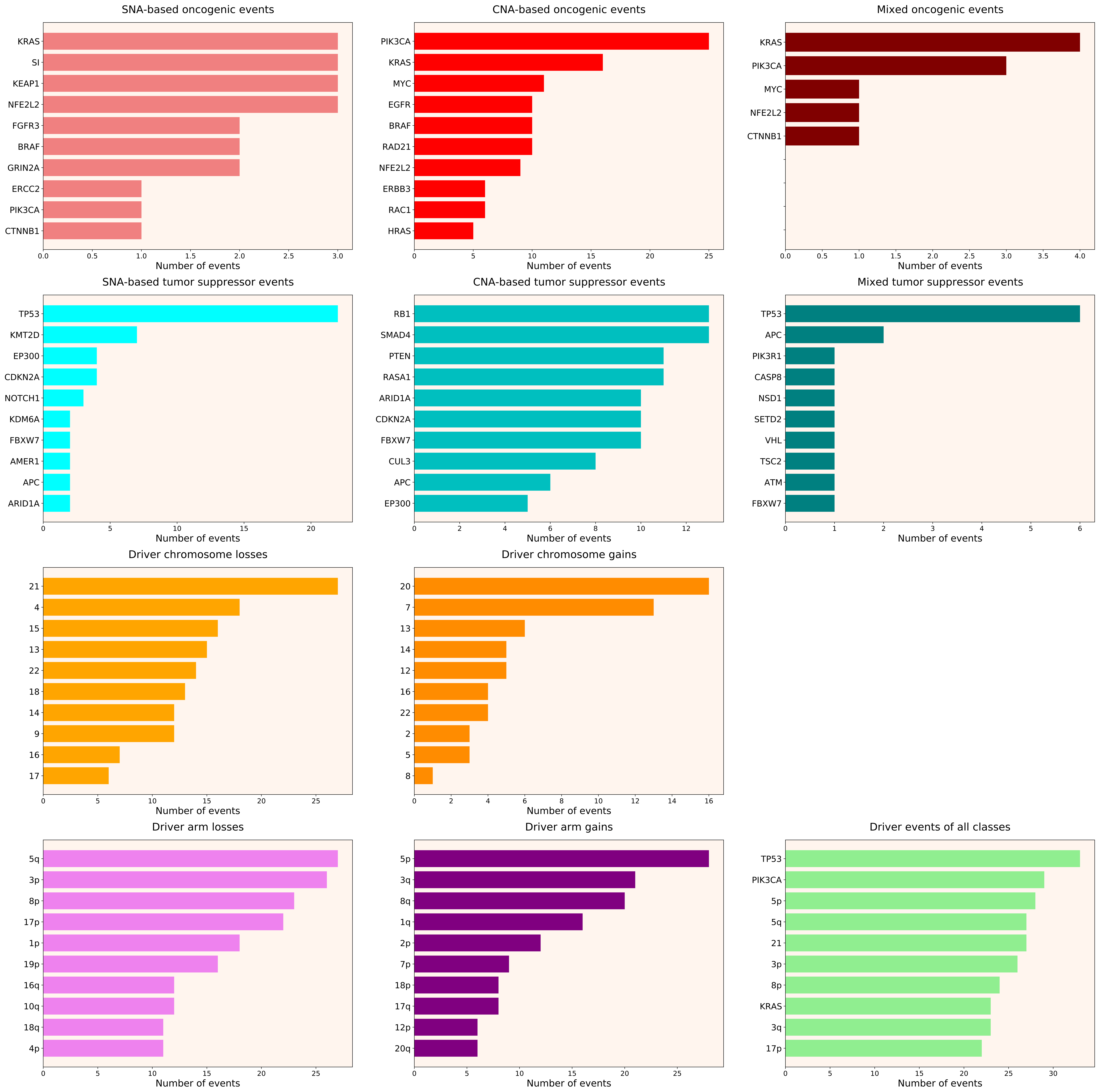
SNA-based oncogenic events
KRAS
SI
KEAP1
NFE2L2
FGFR3
BRAF
GRIN2A
ERCC2
PIK3CA
CTNNB1
0.0 0.5 1.0 1.5 2.0 2.5 3.0
Number of events
CNA-based oncogenic events
PIK3CA
KRAS
MYC
EGFR
BRAF
RAD21
NFE2L2
ERBB3
RAC1
HRAS
0 5 10 15 20 25
Number of events
Mixed oncogenic events
KRAS
PIK3CA
MYC
NFE2L2
CTNNB1
0.0 0.5 1.0 1.5 2.0 2.5 3.0 3.5 4.0
Number of events
SNA-based tumor suppressor events
TP53
KMT2D
EP300
CDKN2A
NOTCH1
KDM6A
FBXW7
AMER1
APC
ARID1A
0 5 10 15 20
Number of events
CNA-based tumor suppressor events
RB1
SMAD4
PTEN
RASA1
ARID1A
CDKN2A
FBXW7
CUL3
APC
EP300
0 2 4 6 8 10 12
Number of events
Mixed tumor suppressor events
TP53
APC
PIK3R1
CASP8
NSD1
SETD2
VHL
TSC2
ATM
FBXW7
0 1 2 3 4 5 6
Number of events
Driver chromosome losses
21
4
15
13
22
18
14
9
16
17
0 5 10 15 20 25
Number of events
Driver chromosome gains
20
7
13
14
12
16
22
2
5
8
0 2 4 6 8 10 12 14 16
Number of events
Driver arm losses
5q
3p
8p
17p
1p
19p
16q
10q
18q
4p
0 5 10 15 20 25
Number of events
Driver arm gains
5p
3q
8q
1q
2p
7p
18p
17q
12p
20q
0 5 10 15 20 25
Number of events
Driver events of all classes
TP53
PIK3CA
5p
5q
21
3p
8p
KRAS
3q
17p
0 5 10 15 20 25 30
Number of events
